# How to deal with ground truthing affected by human‐induced habitat change?: Identifying high‐quality habitats for the Critically Endangered Red Siskin

**DOI:** 10.1002/ece3.3628

**Published:** 2017-12-10

**Authors:** Ada Sánchez‐Mercado, Kathryn M. Rodríguez‐Clark, Jhonathan Miranda, José Rafael Ferrer‐Paris, Brian Coyle, Samuel Toro, Arlene Cardozo‐Urdaneta, Michael J. Braun

**Affiliations:** ^1^ Centro de Estudios Botánicos y Agroforestales Instituto Venezolano de Investigaciones Científicas (IVIC) Caracas Venezuela; ^2^ Centro de Ecología IVIC Caracas Venezuela; ^3^ Provita Caracas Venezuela; ^4^ Smithsonian National Museum of Natural History Washington DC USA

**Keywords:** endangered species, random forest, species distribution models, Venezuela

## Abstract

Species distribution models (SDM) can be valuable for identifying key habitats for conservation management of threatened taxa, but anthropogenic habitat change can undermine SDM accuracy. We used data for the Red Siskin (*Spinus cucullatus*), a critically endangered bird and ground truthing to examine anthropogenic habitat change as a source of SDM inaccuracy. We aimed to estimate: (1) the Red Siskin's historic distribution in Venezuela; (2) the portion of this historic distribution lost to vegetation degradation; and (3) the location of key habitats or areas with both, a high probability of historic occurrence and a low probability of vegetation degradation. We ground‐truthed 191 locations and used expert opinion as well as landscape characteristics to classify species' habitat suitability as excellent, good, acceptable, or poor. We fit a Random Forest model (RF) and Enhanced Vegetation Index (EVI) time series to evaluate the accuracy and precision of the expert categorization of habitat suitability. We estimated the probability of historic occurrence by fitting a MaxLike model using 88 presence records (1960–2013) and data on forest cover and aridity index. Of the entire study area, 23% (20,696 km^2^) had a historic probability of Red Siskin occurrence over 0.743. Furthermore, 85% of ground‐truthed locations had substantial reductions in mean EVI, resulting in key habitats totaling just 976 km^2^, in small blocks in the western and central regions. Decline in Area of Occupancy over 15 years was between 40% and 95%, corresponding to an extinction risk category between Vulnerable and Critically Endangered. Relating key habitats with other landscape features revealed significant risks and opportunities for proposed conservation interventions, including the fact that ongoing vegetation degradation could limit the establishment of reintroduced populations in eastern areas, while the conservation of remaining key habitats on private lands could be improved with biodiversity‐friendly agri‐ and silviculture programs.

## 1 INTRODUCTION

One of the most promising applications of species distribution modeling (SDM) for conservation management is ranking areas by estimated habitat quality (Kramer‐Schadt, Revilla, & Wiegand, 2005). This use of SDM assumes that areas with high probabilities of occurrence predict high‐quality habitats (Franklin, 2010). However, species are not always present where high occurrence probabilities are predicted (Peterson et al., 2011). This mismatch between modeled predictions and field observations may result from problems with the SDM itself, such as conceptual errors (e.g., when models do not include biogeographical barriers or biotic interactions), limitations in variable selection (due a poor understanding of factors driving species distribution or use of outdated presence information with respect to environmental predictors used (Peterson et al., 2011). However, in other cases, this mismatch may be driven by anthropogenic processes such as increased poaching and overexploitation (Sánchez‐Mercado et al., 2014) or land transformation due urban or agricultural development. For these reasons, field validation of SDM predictions is recommended; however, it is often not performed (Greaves, Mathieu, & Seddon, 2006).

The most frequent strategy for validating SDM predictions in the field is searching for the species of concern in areas with high values of predicted occurrence probabilities: Detections are interpreted as a confirmation of high‐quality habitat (Bosso, Rebelo, Garonna, & Russo, 2013; Rebelo & Jones, 2010). However, if land cover transformation is gradual then, species detection is still possible where habitat has been partly degraded but not lost. Such “snapshot detections” of species occurrence may generate a misleading picture of relative habitat quality, which in turn could have disastrous consequences if, for example, the model is used to identify areas for the reintroduction of captive‐bred endangered species (Lahoz‐Monfort, Guillera‐Arroita, & Wintle, 2014). In such situations, a more nuanced, nonbinary approach to field validation is essential. On the other hand, lack of detection can be noninformative if the species is temporarily absent from high‐quality habitat due to seasonal movements or has suffered strong declines due to habitat‐unrelated threats such as poaching or other forms of wildlife extraction.

Clearly, validating SDM based only on species detections could be inappropriate in areas threatened by vegetation degradation and wildlife extraction. We therefore developed a new approach for field validation when a mismatch between model results and simple detection is likely. In this study, we used historical presence records for the Red Siskin (*Spinus cucullatus*), a low‐abundance, Critically Endangered, and little‐studied bird, to examine the combined utility of species distribution models and ground truthing via this new approach, to improve identification of key habitats for conservation interventions. The Red Siskin, a small Neotropical finch, is a particularly appropriate system in which to develop these alternative methods because it has been largely extirpated from its historic range across Venezuela, eastern Colombia, and Trinidad (Rodríguez, García‐Rawlins, & Rojas‐Suárez, 2015). Currently, the species persists in a few isolated populations within Venezuela and in a recently discovered small disjunct population in Guyana (Robbins, Braun, & Finch, 2003). The Red Siskin is listed as Endangered globally and Critically Endangered in Venezuela as a result of historic overexploitation for the specialized pet trade, and captive breeding and reintroduction have been recommended as management interventions; however, habitat loss is thought to be an important threat, although data are scan (Rodríguez‐Clark et al., 2015).The natural habitat of the Red Siskin in Venezuela includes primarily tropical premontane humid and dry forests (Coats & Phelps, 1985) the latter of which are among the most threatened ecosystems globally and are endangered in Venezuela due to conversion for urbanization and agriculture (Rodríguez, Rojas‐Suárez, & Giraldo Herández, 2010). To date, there has been no systematic assessment to determine the amount and location of remaining high‐quality habitat for Red Siskins, nor the threat of deterioration those habitats face.

The conservation action plan for the Red Siskin in Venezuela proposed by the Red Siskin Initiative recommends the eventual restoration of this species via reintroductions into suitable habitat, in areas where the original threats—trapping and habitat loss—have been controlled (http://www.redsiskin.org/). To accurately identify areas that can support viable populations, it is necessary to understand the relationship between Red Siskin habitat requirements and these landscape units, as well as to assess how these units have changed over time. Here, we use a representative dataset of presence records and species distribution models (SDMs) based on maximum likelihood to estimate the historic probability of occurrence of the Red Siskin in Venezuela. Then, we fit a Random Forest classification model (RF) to predict the spatial distribution of current habitat suitability—based on expert ground evaluation—as a response to vegetation degradation. We combined SDM and RF results to examine the drivers of model mismatch, addressing three basic questions about species habitat availability: (1) How extensive was the historic distribution of Red Siskins in Venezuela? (2) How widespread is habitat loss as measured by vegetation degradation? and (3) Where are remaining key habitats, or areas with both high historic occurrence probability and low landscape transformation? In addition, to demonstrating the usefulness of our approach for identifying key habitats for a threatened, elusive, and poorly studied species, we also aimed to examine implications for the threat status of the species and consider the consequences of our results for the design of effective strategies for reintroduction, including habitat conservation, which may be needed to achieve self‐sustaining populations of the Red Siskin.

## 2 METHODS

### 2.1 Study area

Although the precise historical range of the Red Siskin is unknown, expert opinion can be used to identify an appropriate study area that is likely to contain this range. Experts agree that the distribution of this species in Venezuela is shaped principally by three factors: elevation, forest cover, and humidity (Coats & Phelps, 1985; Rivero Mendoza, 1983). The Red Siskin is thought to use a variety of habitats including dry deciduous woodland, mixed deciduous forest, evergreen forest, and the savanna‐forest ecotone with daily and seasonal movements covering several kilometers from humid premontane forest into drier semideciduous forest and grassy clearings. Red Siskins use elevations from 200 to 1500 m (Coats & Phelps, 1985; Hilty, 2003; Rivero Mendoza, 2004). Based on these expert habitat descriptions, our approach for generating the study area polygon was based on estimating the Extent of Occurrence using the overlap of three environmental variables as proxies for factors describing suitable Red Siskin habitat: (1) elevation model (ELEV, 1 km of resolution; CGIAR Institute, 2010); (2) proportion of tree cover (TREE, 0.5 km of resolution; Hansen et al., 2002); and (3) aridity index (AI, 1 km; Zomer, Trabucco, Bossio, van Straaten, & Verchot, 2008).

Elevation values in Venezuela range from 0 to 4,382 m; we created a binomial layer defining as suitable (1) range from 200 to 1500 m, and values outside this range as unsuitable (0). Tree cover values in northern Venezuela ranged from 0% to 84%; because Red Siskins can use several vegetation types with different coverage, we used a broad threshold so that we could include a wider range of vegetation cover. Thus, we defined as suitable tree cover values (TREE) from 10% to 100%. Aridity index (AI) represented precipitation availability over atmospheric water demand, the ratio of mean annual precipitation, and potential evapotranspiration, between the years 1950 and 2000. AI values ranged from 1,847 to 26,349, with higher values representing more humid conditions. We defined AI values from 2,000 to 6,500 to be suitable, which corresponded to semiarid and dry subhumid conditions (Trabucco & Zomer, 2009; Zomer et al., 2008).

For the study area definition and for the analysis of historical probability of occurrence (see below), we decided to change the spatial resolution of all predictive variables to the lowest spatial resolution (1 km) to minimize error due to spatial uncertainty in georeferencing historical distribution records (Wieczorek & Hijmans, 2004). For that, we aggregated (decreasing the resolution) using nearest‐neighbor resampling method (by centering). This method is implemented in the function *resample* from *raster* package in R (Hijmans & Van Etten, 2012).

**Figure 1:**
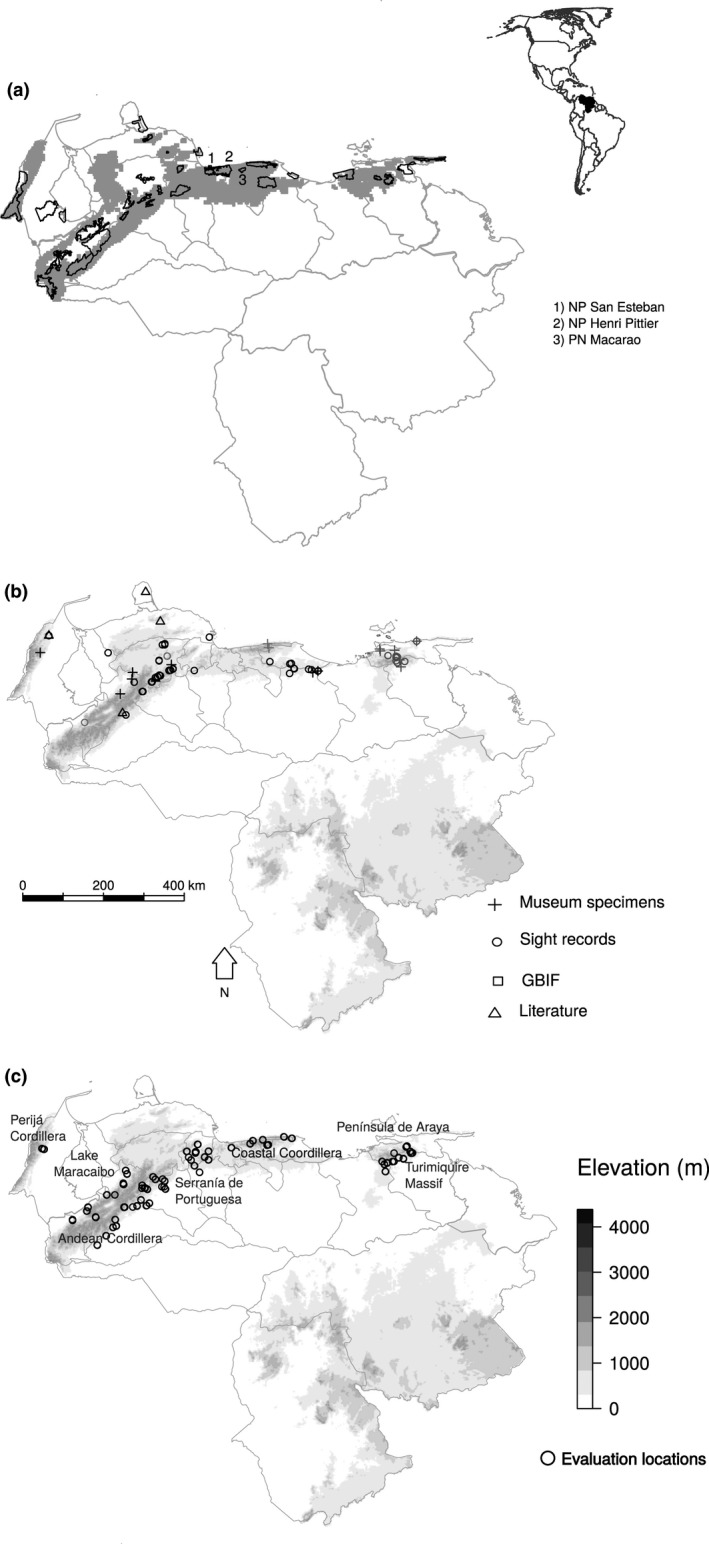
(a) Map of the study area in Venezuela. Gray lines represent political divisions; gray polygons are the study area for habitat and historical occurrence; black lines enclose national parks. The most relevant national parks are labeled. (b) Elevation layer with Red Siskin presence records compiled from different sources of data. Gray symbols indicate records before 1960; black symbols indicate records after this year. (c) Elevation layer with locations in which habitat quality was evaluated. The most important geographic features are labeled

Finally, we overlapped all binomial layers to define a study area within which minimal habitat conditions for Red Siskins were met (90,060 km^2^; Figure 1a).

### 2.2 Historical probability of occurrence

We estimated the historical probability of occurrence (Ψ_H_) for the Red Siskin within the study area using a compilation of historic and recent presence records and models based on maximum likelihood. We first made an extensive search for historic and current records of species presence from four sources: (1) Global Biodiversity Information Facility (GBIF) (2) national and international museums, (3) interviews with local ornithologists, and (4) a literature review. For all sources, we used keywords in English and Spanish related to all common and scientific names of the species (considering synonyms, alternative spellings, and subspecies) using the list compiled by Encyclopedia of Life (www.eol.org): Black Hooded Siskin*, Cardenalito, Cardenal, Carduelis cucullata,* Red Siskin, *Spinus cucullatus, Sporagra cucullata*.

From GBIF, we obtained 26 records from 1898 to 2010 (most from 1898; GBIF Secretariat, 2016). We reviewed the bird collections from five international museums (American Museum of Natural History, Yale Peabody Museum of Natural History, Smithsonian National Museum of Natural History, Royal Ontario Museum, and British Museum of Natural History), and three local museums (Colección Ornitológica Phelps, Museo de Barquisimeto, and Museo de Biología de Rancho Grande). From these museums, we compiled 92 records of specimens collected from 1847 to 2008. Additionally, 43 presence records of Red Siskins observed between 1995 and 2013 were reported during interviews with six local ornithologists with long‐term experience in Venezuela: Chris Sharpe, Fidel Escola, Gustavo Rodríguez, José Gustavo León, Jhonathan Miranda, and David Ascanio.

Finally, we did a systematic review of the scientific literature using ISI Web of Knowledge and Google Scholar, using keys words related to the species and found seven published works (Coats, 1982; Coats & Phelps, 1985; Collar et al., 1992; López, 1991; Phelps & Phelps, 1963; Rivero Mendoza, 1983, 2004) from which we retrieved 332 records from 1867 to 1992. In total, we compiled 491 records of species presence from 1847 to 2013 (Figure 1b).

We considered a record to be any discrete observation of one or more birds with a unique combination of the following information: (1) source, (2) coordinates, (3) observation year, (4) sex and development stage (adult or juvenile), and (5) quantity reported (number of individuals). For records without specific geographic coordinates, we used location descriptions (place names, geographic features, etc.) to assign latitude and longitude based on gazetteers (GIS Data Depo, DIVA GIS). When contrasting coordinates were provided by each gazetteer, we calculated the mean value and error of latitude and longitude. If the error was larger than the cell resolution used to project our predictions (1 km^2^) or if original coordinates had rounded two decimals, we discarded the record (Figure 1b).

To estimate the historical probability of occurrence (Ψ_H_) for the Red Siskin, we used a maximum likelihood approach based on logistic regression to fit a species distribution model as a function of covariates, as implemented in R (“MaxLike”; Royle, Chandler, Yackulic, & Nichols, 2012). In addition to the three covariates used to define the study area, we also considered the 19 climatic variables in the WorldClim dataset as predictors (resolution 1 km; Hijmans, Cameron, Parra, Jones, & Jarvis, 2005). We evaluated redundancy and collinearity between all covariables using a hierarchical cluster analysis based on Pearson correlation. We defined a cluster as the group of variables with correlation <0.6 and selected one covariable for each cluster (Sarle, 1990). Thus, our complete model included the following eight variables: aridity index (AI), forest cover (TREE), mean diurnal temperature range (BIO02), isothermality (BIO03), annual temperature range (BIO07), mean temperature of the warmest quarter (BIO10), precipitation in the driest quarter (BIO17), and precipitation in the coldest quarter (BIO19). We applied a square‐transformation to variables with considerable skew (TREE*,* BIO03, BIO10, BIO17, BIO18, and BIO19) and standardized all variables to a zero mean and unit variance, as recommended for the algorithm implemented (Royle et al., 2012).

To fit the occurrence model, we used only the 88 georeferenced presence records from 1960 or later, because our covariates were built with data from this date or later. The MaxLike approach assumed that detectability was constant over the study area (Royle et al., 2012). Sampling effort was extensive enough over time (56 years) to have detected the species in the areas considered if it was present, leaving detection probability sufficiently uniform to meet this assumption. However, MaxLike also assumed that sampling was random, which clearly was not the case, even though our dataset included almost all known sources of records. Thus, to surmount this problem, we applied a random sampling to the reports and repeatedly partitioned them into two independent subsets: an occurrence probability calibration subset and an occurrence validation subset (see details below; Franklin, 2010). The calibration subset was used to fit SDMs as described below and consisted of 66 reports (75% of the data). The validation subset (22 reports) was used to validate SDM performance. We repeated this two‐way partitioning five times, which created replicates allowing us to directly evaluate data heterogeneity (Peterson et al., 2011).

To select the “best” MaxLike occurrence probability model, we then fit different combinations of the eight covariates described above to each of the five replicate calibration data subsets. Our first model (mdl1) contained linear terms for all eight variables. The second (mdl2) included only climatic variables, and the third model (mdl3) was the most reduced, including only forest cover and aridity index, which have been proposed by experts to be the most important variables affecting Red Siskin occurrence (Rivero Mendoza, 2004). The “best” model was considered to be the one that both converged and had the lowest AICc in the most replicates (Table 1; Burnham & Anderson, 2002).

**Table 1:** Statistical support (AICc values), and convergence status for three models of red siskin occurrence, fit with MaxLike to five replicate data subsets

Model	Replicate	AICc	Convergence
mdl1 = TREE + AI + BIO02 + BIO03 + BIO07 + BIO10 + BIO17 + BIO19			
	1	647.982	No
	2	647.691	No
	3	635.865	No
	4	635.463	No
	5	695.351	No
mdl2 = BIO02 + BIO03 + BIO07 + BIO10 + BIO17 + BIO19			
	1	650.621	No
	2	642.334	No
	3	633.761	No
	4	634.553	No
	5	692.630	No
mdl3 = TREE + AI			
	1	696.733	Yes
	2	676.306	Yes
	3	677.510	Yes
	4	678.468	Yes
	5	738.753	Yes

We used *evaluate* and *threshold* functions from *dismo* package in R (Fielding & Bell, 1997) to (1) select the replicates of the best model with the best performance to built spatial prediction, and (2) select the threshold of historic probability of occurrence at which Red Siskin presence is the highest (*T*_ΨH_). Model prediction was evaluated based on correlation coefficient (cor), Area Under the Receiver Operator Curve (AUC), and maximizing the sum of sensitivity and specificity (maxSSS) using as pseudoabsences 88 points that were randomly selected from the northern part of the country, but outside of the study area for the Red Siskin. We selected the replicates 1, 2, and 5, which had the highest values of AUC, cor, and maxSSS to built the spatial prediction (Table 2). We used the mean value of statistic “max kappa” (predicted value at which kappa is highest; Liu, Berry, Dawson, & Pearson, 2005) of selected replicates as criteria to set *T*_ΨH_ (0.743). To built the spatial prediction, we used the *predict* function of *maxlike* package (Royle et al., 2012) and a raster stack of the same predictive variables disaggregated at a resolution of 250 m to produce a map that were comparable with our other predictions (see below).

**Table 2:** Performance indices for the best model of Red Siskin occurrence (mdl3), fit with MaxLike to five replicate data subsets

	Replicate 1	Replicate 2	Replicate 3	Replicate 4	Replicate 5
Number of presence records	17	18	17	18	17
Number of absences records	70	70	70	70	70
AUC	0.770	0.733	0.734	0.664	0.747
cor	0.374	0.346	0.333	0.210	0.333
maxSSS	0.589	0.391	0.240	0.103	0.240
Max kappa	0.786	0.692	0.544	0.139	0.751

AUC = area under the curve of Receiver Operating Characteristic. cor = correlation coefficient. maxSSS = maximizing the sum of sensitivity and specificity. Max kappa = prediction value at which kappa statistic is the highest.

### 2.3 Ground‐truthing and vegetation time series analysis

We randomly selected 90 points within the study area (Figure 1c) to perform ground truthing of habitat suitability. At each point, we walked transects of 1.5 km, which we laid on the roads nearest to the point. Along each transect, we stopped every 500 m, resulting in 270 “evaluation locations” in total (90 random points * 3 stops per point). However, several of these evaluation locations were in areas that were either inaccessible or in areas with high risk to the personal security of field teams. We, therefore, systematically discarded 79 locations present in dangerous or inaccessible areas, resulting in a total of 191 evaluation locations.

From July 2015 to March 2016, two of us, including an expert ornithologist with 5 years' experience studying Red Siskins in the field (JM) and an experienced assistant (ST), visited each evaluation location and classified it into one of four categories of habitat suitability for Red Siskin breeding (excellent, good, acceptable, poor), based on geographic characteristics (elevation, slope), and vegetation type (forest, shrubs, and pasture) that are considered important according to expert accounts and own field experience (Coats & Phelps, 1985; Rivero Mendoza, 1983, 2004). Locations with “excellent” suitability were those with mosaic of forests surrounded by shrubs, located at medium elevations (500–800 m) and steep slopes (>60%). Locations with “good” suitability had forest surrounded by shrubs, but also by pastures or crops, had medium elevation and moderate slopes (20–30%). “Acceptable” areas were composed mostly of shrubs and pastures at low elevations (200–400 m) and moderate slopes. Finally, “poor” suitability areas consisted of transformed vegetation (pastures, urbanized areas) at low elevations (<100 m) and slopes <10%, or fully forested areas with no surrounding shrubs or fields, at extremely steep slopes. At each evaluation location, one person recorded landscape characteristics while the Red Siskin expert categorized overall habitat suitability for species breeding. We then graphically examined the agreement between expert classification and landscape characteristics evaluated (Fig. S1).

We next evaluated the accuracy and precision of habitat suitability categories by relating them with recent Enhanced Vegetation Index time series (EVI). We used the MODIS Vegetation Index Product Series Collection 5 (MOD13Q1, version 5; Land Processes Distributed Active Archive Center ‐ LP DAAC, 2014), which is available at 250 m of spatial resolution, and 16‐day of temporal resolution (from February 2000 to June 2015). EVI measures chlorophyll concentration in canopy vegetation and permits meaningful comparisons of seasonal and interannual changes in vegetation growth and activity (Huete et al., 2002). The MODIS EVI product is computed from atmospherically corrected bidirectional surface reflectances that have been masked for water, clouds, heavy aerosols, and cloud shadows (Land Processes Distributed Active Archive Center – LP DAAC, 2014). The EVI value (where 0 indicated no vegetation and 1 indicated vegetation saturation) is the result of “compositing” algorithm in which, several EVI images, over 16 days time interval, are merge to create a single cloud‐free image EVI map with minimal atmospheric and sun‐surface‐sensor angular effects.

We used the quality assurance flags (MODLAND_QA) to rank EVI observations (combination of localities and time periods): 37% of the observation had high quality, 47% had median quality, and 16% had poor quality (Land Processes Distributed Active Archive Center (LP DAAC), 2014). For all the analysis, we used the mean values of the observations with the highest quality available and discarded poor‐quality observations unless necessary.

In order to relate the subjective habitat suitability classification with the measured EVI phenology, we fit a random forest classification model (RF). We implemented RF in the *randomForest* package in R (Liaw & Wiener, 2002). For each ground‐truthing location, we coded the expert's suitability assessment as an ordinal variable with four categories and used the 23 values of the EVI phenology as explanatory variables. We built each classification tree with a training dataset containing 63% of records sampled randomly with replacement from the original data and containing a random subset of five predictor variables selected from the full set of predictor variables. We resampled records to create 50,000 classification trees in our RF. To evaluate the classification power of RF, we used the remaining 40% of records (i.e., “out‐of‐bag” observations, OOB). An estimate of the misclassification error rate was calculated for each OOB observation and averaged over all trees in the forest (Cutler, Edwards, & Beard, 2007). As the response variable was an ordinal variable, the OOB confusion matrix could overstate the classification error of the final RF model between contiguous categories. Therefore, we applied a matrix of ordered weights to recalculate the OOB (Piccarreta, 2008).

We visualized the spatial distribution of habitat with current optimal suitability using the *predict* function of *randomForest* package (Liaw & Wiener, 2002) and a raster stack of predictive variables (EVI 16‐day values for the year 2014) at a resolution of 250 m. Due to the inherent uncertainty in subject classifications and the resulting high RF classification error, we transformed the outcome of the prediction from a matrix of RF votes per category into a numerical value per pixel using single‐step category weights (0 for “poor,” 3 for “excellent”). The resulting map represents an index based on the weighted average of habitat suitability predictions in each 250 m cell.

EVI also allows the meaningful comparison of seasonal and interannual changes in vegetation growth and activity. We therefore used the time series of EVI data to describe changes in the mean and variance of EVI values within the period studied at each evaluation location. Changes in local vegetation did not occur simultaneously for all evaluation locations. Thus, we used the method proposed by Chen and Gupta (2000) to estimate the most likely point of significant change in mean and variance in each time series (i.e., the change point), as implemented in the function *cpt.meanvar* from package *changepoint* in R (v 2.2.1; R Development Core Team, 2015). We compared mean EVI values before (prior mean EVI) and after (current mean EVI) the change point for each series, but considered only the current values to calculate the EVI phenology (multi‐year median values for each 16‐day period) at each site.

### 2.4 Identifying key habitats

Finally, we overlapped the historic probability of occurrence (from our SDM) and current habitat suitability predictions (from our RF) to identify “key habitats,” or areas with both high historic occurrence probability and excellent habitat suitability. To identify key habitats, we multiplied the values of both predictions to generate an overlap index that ranged from 0 to 2.248, with 0 indicating low historic probability of occurrence and low habitat quality and values close to 2 indicating high values for both conditions. We also performed an extinction risk assessment using the criterion of population reduction based on an estimated decline in Area of Occupancy (AOO) and habitat quality (criterion A2c; IUCN, 2012). We estimated AOO based on the number of cells containing key habitats and calculated the percentage of reduction in this area with respect to the historically suitable area for Red Siskins (i.e., areas with Ψ_H_ > 0.743). We also calculated AOO when key habitats were defined to include both “good” and “excellent” habitat suitability, to take into account uncertainty in Red Siskin habitat use.

## 3 RESULTS

### 3.1 Spatial distribution of habitat with current optimal conditions

**Figure 2:**
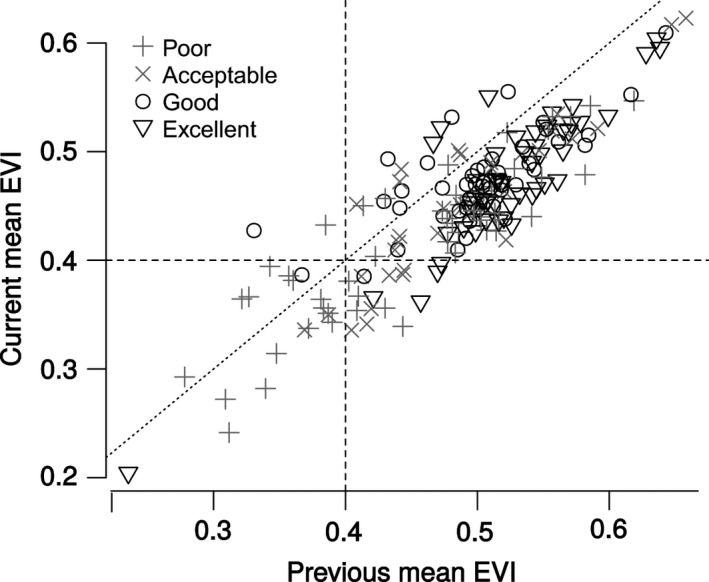
Changes in EVI during from 2000 to 2015 for each evaluation location within the Red Siskin study area. The abscissa indicates the mean EVI value before the inflection point defined for each evaluation location. The ordinate reflects the mean EVI value after the inflection point. The four habitat quality classes are indicated

The majority of the evaluation locations (85%) had a substantial reduction in mean EVI values over the last 15 years (Figure 2). These changes were similar across different categories of habitat quality (chi‐square = 4.707; *df* = 3; *p* = .195). For 60% of the evaluation locations, this change occurred before 2011. Locations classified with optimal habitat suitability (“good” and “excellent”) consistently had historic mean EVI values >0.4. Although for these same locations, current mean EVI values were substantially lower, they were still above 0.4. Locations classified with suboptimal habitat suitability (acceptable and poor) were more heterogeneous, with EVI values from 0.3 to 0.6, but mostly below the mean values of the optimal habitats (Figure 2).

**Figure 3:**
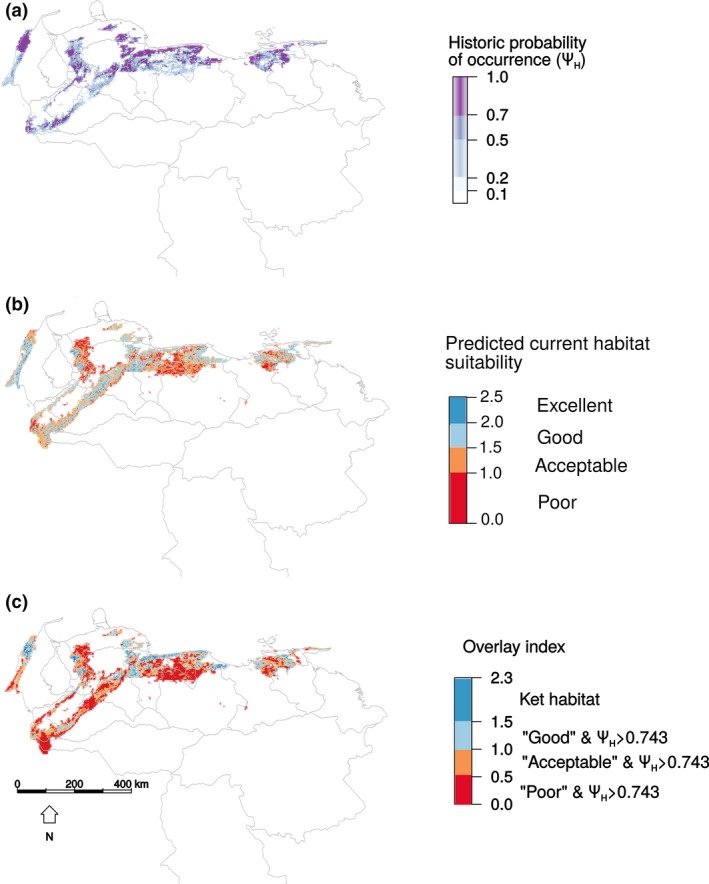
(a) Spatial distribution of the current habitat quality predictions based on EVI time series and the random forest classification model. (b) Historic probability of occurrence for Red Siskins in Venezuela derived from replicates of the best performing MaxLike model. (c) Overlap between historic occurrence probability and current habitat quality. Gray lines represent political boundaries in each panel

The overall corrected classification error rate of the RF model was 33.2%, with lowest classification error for “excellent” (22.1%) largest for “acceptable” (57.1%). Habitats classified as “good” were predicted in a wide area within the study area (35,494 km^2^, 39% of study area), while “excellent” habitat (3,127 km^2^, 3% of study area) were clustered in the western part of the country, in the lowlands of the Sierra de Perijá and along the southern slope of the Cordillera de Mérida (Figure 3a). Other small and more dispersed blocks of “excellent” habitat were predicted in the center‐west as well as in the east. Habitat with suboptimal conditions (“poor” and “acceptable”) was focused in three large blocks (55,307 km^2^, 61%) in the west, center, and east of the country.

### 3.2 Historical probability of occurrence

Presence records before 1960 (25 records) were located mostly in the western part of the country, while more recent sightings (94) were evenly distributed across the center and west (Figure 1b).

The best model for historic probability of occurrence was mdl3 (containing only the aridity index and forest cover). Alternative models containing other climatic variables did not converge due to scarcity of records (Table 1). Our estimates of historical occurrence probability were based on the three replicates of mdl3 that had good predictive accuracy (Table 2). The area with the highest occurrence probabilities (Ψ_H_ > 0.743) covered 20,696 km^2^ and was concentrated toward the center and north of the Coastal Cordillera. In the west, small fragments with high probability were observed toward the south, along the north slope of the Andean Cordillera and Serranía de Portuguesa, and along the eastern coast of Lake Maracaibo (Serranía del Empalado). To the east, there were also discontinuous fragments around the Turimiquire Massif and west of the Araya Península (Figure 3b).

Of the area with the best historically suitable area for Red Siskins (Ψ_H_ > 0.743), only 4,686 km^2^ (23%) was protected in a Venezuelan national park. The most valuable unprotected habitats were in the western region, including areas in the northern Sierra de Perijá, along the west coast of Lake Maracaibo and in the mountains of Falcón and Lara. In the northeast, there was also a wide continuous area with high probabilities on unprotected lands (Figure 3b).

### 3.3 Key habitats

Key habitats, defined as areas with both high historic occurrence probabilities and currently “excellent” suitability, covered just 976 km^2^ and occurred in the western and central regions (northern end of the Sierra de Perijá, lowlands of Sierra de El Empalado, and the Coastal Cordillera), forming small blocks (Figure 3c). Only three small national parks (San Esteban, Macarao, and Henri Pittier) and one natural monument (Pico Codazzi), included portions of these key habitats (279 km^2^), while the remaining areas did not have protected status. If key habitats were expanded to include both “good” and “excellent” areas, their area increased by an order of magnitude, to 12,274 km^2^.

If the present Area Of Occupancy (AOO) was presumed to consist of key habitats (976 km^2)^, this represented a decline of 95% of the historic range (20,696 km^2^ with Ψ_H_ > 0.743). This decline corresponded to the risk category of Critically Endangered (A2c; IUCN, 2012). In contrast, if expanded key habitat (12,274 km^2^) was considered, the decline was 40%, which corresponded to the risk category of Vulnerable.

## 4 DISCUSSION

### 4.1 Key habitats

Our approach of combining species distribution models and Random Forest models proved to be useful in revealing substantial mismatch between historical predictions and present conditions, and identifying key habitats for Red Siskin conservation, or areas with historically high occurrence probabilities and currently optimal habitat suitability, for Red Siskin conservation. We were able to identify areas with suitable environmental and ecological conditions for species occurrence, as well as areas where ongoing land transformation has negatively affected the species' historical habitat. There is a high probability of finding new populations of Red Siskin within the 20,696 km^2^ with the highest predictive scores for historical occurrence. However, vegetation degradation within threatened habitats, such as dry forests (Rodríguez et al., 2010), has likely further reduced the area of suitable habitats for this species. For the Red Siskin, this reduction represented a loss between 40% and 95%, resulting in a IUCN category between Vulnerable and Critically Endangered for criterion A2c (IUCN, 2012). Given the scarcity of records throughout this range, it is furthermore possible that the actual area occupied by the species is far less than the area available. This is likely because we hypothesize that in addition to habitat loss, we suspect the Red Siskin has suffered what is known as a “high‐abundance‐biased” or HAB decline (Rodríguez, 2002): Individuals were likely removed from the geographic range not randomly or evenly from across the range, but rather in a way that was biased toward high‐abundance areas. This is because trappers seem to have been specialized, and interested in this species in particular, and so likely searched for and trapped it precisely in the areas that they were most likely to find it.

Our analysis provides the first quantitative evidence that in addition to overexploitation, land transformation may also be driving the extirpation of Red Siskins in Venezuela, and also reveals that ongoing habitat transformation could limit the establishment of reintroduced populations there (Figure  3a and c). The distribution of key habitats corresponded well to the conservation status described by Coats and Phelps (1985): The western region had a greater extent of suitable habitat than the central region and may harbor the largest remaining populations, while populations of eastern Venezuela seem to have been extirpated. Coats and Phelps attributed this extirpation of eastern Red Siskin populations to extensive exploitation for the cage bird trade. Indeed, trappers have reported thousands of individuals smuggled from eastern Venezuela to the nearby island of Curacao (Coats & Phelps, 1985) and unsustainable trapping may still occur in this region (Dessene & Strahl, 1991; Marín‐Espinoza, Guevara‐Vallera, Prieto‐Arcas, Muñoz‐Gil, & Carvajal‐Moreno, 2011). However, these areas are also the ones most affected by vegetation change. Our estimated large reduction in suitable areas for Red Siskins reflects years of constant land transformation, which implies a generalized degradation of vegetation, affecting habitats across the board, regardless of their suitability as Red Siskin habitat. Although habitats with optimal conditions have thus far retained some forest cover (EVI values above 0.4), the ongoing degradation observed suggests that remaining blocks with optimal habitat conditions could also be degraded in the short‐to‐medium term, reducing the availability of suitable habitat for this critically endangered species even more.

Beyond the small size and ongoing degradation of key habitats for the Red Siskin, the fact that only a fraction of their area is under protection is relevant to the potential success of future conservation efforts (Figure 3c). Of the 25 protected areas that lie within the study area, just six protected Red Siskin habitats with high historic occurrence probability (Tapo Caparo, Henri Pittier, Macarao, San Esteban, Guatopo National Parks and Pico Codazzi National Monument). However, only three protected areas in the central region included a small proportion of presently key habitats (Henri Pittier, Macarao, and San Esteban National Parks; Figure 1a and 3c). Interestingly, the objective delineation of key habitats described here also helps bring into focus opportunities for conservation action. For example, between Henri Pittier and Macarao National Parks, a potential corridor includes 200 hectares currently covered with shade coffee farms. This agroforestry habitat currently faces an uncertain future due government price restrictions that make standard coffee production unprofitable (SUNDE, 2015). The Red Siskin Initiative has proposed to apply a proven market‐based approach, Bird Friendly Coffee^®^ certification (BFC) to these shade coffee farms, which would qualify their products as a specialty coffee, free of price restrictions (Philpott, Bichier, Rice, & Greenberg, 2007). BFC certification could be a means to protect and improve the shade coffee farm habitat present in this corridor, preserving a potential reintroduction site for Red Siskins that is also prime habitat for migratory birds.

### 4.2 Model accuracy

The Random Forest model is used here to transform a subjective evaluation of habitat quality into an spatial index of habitat suitability for conservation planing. This application assumes that the categories suggested by experts are indeed predictive of the occurrence and viability of the species in the field, and the selected variables are good indicators of the expert ranking. This is, however, a difficult task, given the inherent uncertainty in expert opinions and the natural variability in environmental conditions. The accuracy of the RF model in predicting habitat suitability categories based on EVI time series suggested a moderate overall performance. However, the model was better at discerning optimal than suboptimal habitat conditions. The largest classification errors occurred in habitats with “acceptable” categories, which covered a wide type of vegetation conditions. These errors could be due to a lack of understanding of habitat requirements for such a rare and little‐studied species, which could generate an underestimation of the amount of habitat available to Red Siskins. However, this error could also reflect the capacity of Red Siskins to use transformed habitats, such as ecotones of dry deciduous woodlands, shrubby grasslands, and pastures (Robbins et al., 2003). The widespread overlap between habitats with “acceptable” habitats and areas with high values of historic probabilities of occurrence also supports the idea that Red Siskins are able to use transformed landscapes. This result underlines the importance of conservation actions that reconcile the presence of Red Siskin with transformed landscapes. Initiatives that encourage biodiversity‐friendly agriculture, under a framework of ecoagricultural landscape management (Scherr & McNeely, 2008) might be more successful that the traditional paradigm of protected areas, where agricultural production and other human activities are segregated from areas managed for biodiversity conservation. Clearly, achieving integration between human activities and conservation objectives for Red Siskins in Venezuela requires a more detailed understanding of temporal and spatial patterns of species habitat use. Even more importantly, this will require building capacity for rural communities to adopt biodiversity‐friendly land management (e.g., water source protection, healthy soil management, sustainable agroecology) and the promotion of policies that encourage them (Brussaard et al., 2010).

Our approach has proven useful for identifying key habitats for a threatened and poorly sampled species and also to monitor temporal and spatial trends in vegetation transformation within these habitats. In the case of the Red Siskin, this approach is only the first step toward identifying suitable habitat for reintroduction, which should be refined with additional research focused on breeding and feeding ecology, seasonal movements, and the spatial distribution of poaching risk (e.g., Sánchez‐Mercado, Asmussen, Rodríguez‐Clark, Rodríguez, & Jedrzejewski, 2016).

## CONFLICT OF INTEREST

None declared.

## AUTHOR CONTRIBUTIONS

ASM, KMRC, and JRF conceived the ideas; JM, ST, and ACU collected the data; ASM and JRFP analyzed the data; and ASM, BC, MB, and KMRC led the writing.
