# Supplementary figures and images for: How to deal with ground truthing affected by human‐induced habitat change?: Identifying high‐quality habitats for the Critically Endangered Red Siskin
Source: Ecol Evol. 2017 Dec 10;8(2):841–51. doi: 10.1002/ece3.3628 (PMC5773307; doi:10.1002/ece3.3628)

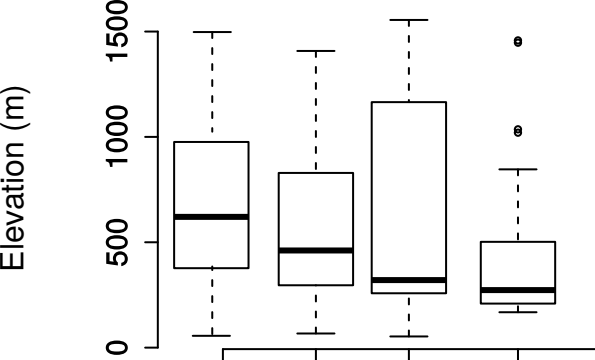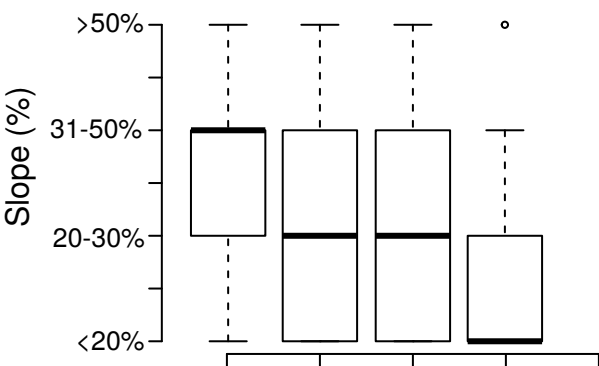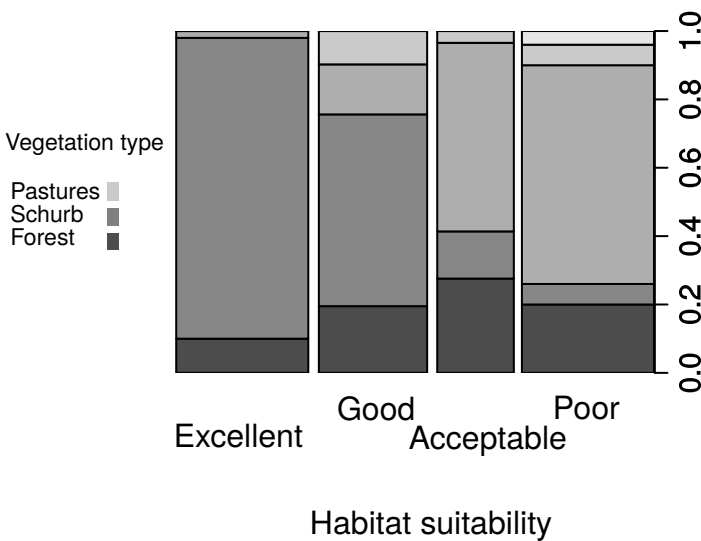

Supplement: Supplementary file 1 [file ECE3-8-841-s001.pdf]
